# Exploring the level of knowledge related to ectopic pregnancy among married Saudi women

**DOI:** 10.3389/fgwh.2026.1714772

**Published:** 2026-01-23

**Authors:** Wjdan Almutairi, Amel Dawod Kamel Gouda, Shujun Almutiri, Rana Alhashemi, Razan Almubaraki, Hanadi Alshehri, Fawziah Almadhabri

**Affiliations:** 1 King Abdullah International Medical Research Center, Riyadh, Saudi Arabia; 2 College of Nursing, King Saud bin Abdulaziz University for Health Sciences, Riyadh, Saudi Arabia; 3 Ministry of National Guard-Health Affairs, Riyadh, Saudi Arabia; 4 Faculty of Nursing, Maternal and Newborn Health Nursing, Cairo University, Cairo Governorate, Egypt

**Keywords:** ectopic pregnancy, knowledge, married women, maternal health, reproductive health

## Abstract

**Background:**

Ectopic pregnancy (EP) is an abnormal condition in which blastocyst implantation occurs outside the lining of the uterus and is the leading cause of pregnancy-related death. Women who have had one ectopic pregnancy are at increased risk. Early diagnosis may reduce the risk of fallopian tube rupture.

**Aim:**

This study assessed the level of knowledge related to ectopic pregnancy among married Saudi women in Riyadh, Saudi Arabia.

**Method:**

A descriptive cross-sectional design was used. This study was conducted at outpatient obstetrics clinics at the Women's Health Specialist Hospital at King Fahad National Guard Hospital in Riyadh. The convenience sample consisted of 255 pregnant women. The data were collected through a structured interviewing questionnaire, which consisted of demographic data and an ectopic pregnancy knowledge assessment tool.

**Results:**

The study's findings revealed that the mean age of the women was 35.50 ± 6.45 years. More than half of the study sample (66.7%) had a university education, and 63.9% delivered via normal vaginal delivery, whereas 27.8% delivered via cesarean section. More than half of the sample had a poor level of knowledge regarding signs and symptoms, diagnosis, complications, and management of EP, with the total mean knowledge score for ectopic pregnancy being 56.96 ± 17.09. Overall, the study sample (60.40%) demonstrated a poor level of knowledge, 31% had a fair level of knowledge, and only 8.6% had a good level of knowledge.

**Recommendations:**

Designing an educational program for women to increase awareness of ectopic pregnancy, including its signs, symptoms, and risk factors, to improve the understanding and prevention of complications of EP.

## 1 Introduction

Ectopic pregnancy is a medical emergency that increases the risk of maternal morbidity and death. It is also the primary cause of maternal mortality in the first trimester of pregnancy, accounting for approximately 10% of all pregnancy-related deaths (1). Pregnant women are prone to high morbidity and mortality due to the undiagnosed and delayed complications of ectopic pregnancy (2). Ectopic pregnancy is an abnormal condition in which the implantation of a blastocyst occurs outside the lining of the uterus. It is particularly important in gynecology in developing countries, as it contributes substantially to first-trimester morbidity and maternal mortality (3). Due to the nonspecific nature of its presentation, ectopic pregnancy should be ruled out first in all pregnant women presenting with abdominal pain, vaginal bleeding, or non-specific symptoms such as nausea and vomiting. Notably, approximately one-third of women with ectopic pregnancy may be asymptomatic, and up to 10% may exhibit no clinical signs at all (4). The clinical indicators that may suggest ectopic pregnancy include increased human chorionic gonadotropin, vaginal bleeding of varying amounts, abrupt lower abdomen pain, pelvic pain, a painful cervix, an adnexal mass, or adnexal tenderness (1). However, adnexal tenderness may only be present when the ectopic pregnancy is located in the adnexa. Clinicians should also consider non-tubal ectopic pregnancies, such as intrauterine ectopic gestations (5).

A successful intrauterine pregnancy depends on a complex interplay of chemical, hormonal, and anatomical factors during fertilization, tubal transport, and embryo implantation (6). Disruption of these processes may predispose to ectopic implantation, and local factors such as prior infection, immunologic alterations, viral exposure, toxic substances, fibrotic, or mechanical mechanisms, thereby delaying embryo transport and increasing the risk of implantation outside the endometrial cavity (7). Ectopic pregnancy presents in several forms, including tubal, corneal, cesarean scar, cervical, heterotypic, abdominal, and ovarian pregnancies. Among these, tubal ectopic pregnancies are the most common and have high maternal morbidity and fatality rates when ruptured. Without timely assessment using the human chorionic gonadotrophin (hCG) or the ultrasound, severe vaginal bleeding from a ruptured ectopic pregnancy may be mistaken for a miscarriage, potentially delaying critical treatment. While symptoms such as nausea, vomiting, and diarrhea are less common in ectopic pregnancy, rupture often precipitates acute clinical manifestations, including hypovolemic shock, peritonitis, abdominal distension, and severe abdominal pain (1).

To treat ectopic pregnancy, confirmation of ectopic pregnancy is necessary. In cases where the early diagnosis of an ectopic pregnancy is unsuitable for active monitoring, medical treatment with methotrexate may be suggested. It is administered intramuscularly and may be given as a single dose or as a multi-dose regimen, depending on clinical criteria, ultrasound findings, initial human chorionic gonadotropin (hCG) levels, and the patient's response to treatment (8). Additionally, laparoscopy, or keyhole surgery, is performed to remove the pregnancy before it becomes too large (8). Early detection of an ectopic pregnancy is essential for preventing additional damage to the tube, lowering the risk of morbidity, and maintaining fertility. An ectopic pregnancy needs to be ruled out if the pregnancy test is positive and the ultrasound reveals an empty uterus (9).

Ectopic pregnancy is reported to be a common condition among women of childbearing age. The reported estimated incidence of ectopic pregnancies in the United States is 1%–2%, and ruptured ectopic pregnancies are responsible for 2.7% of pregnancy-related deaths (10). From 2010 to 2019, there were 15,537 ectopic pregnancies out of 979,027 pregnancies among women in California aged 15–44 years (11). In Saudi Arabia, the reported prevalence of ectopic pregnancy ranges from 1% to 2% of the overall number of pregnancies in Majmaah city of Saudi Arabia (12). Regarding the level of knowledge of ectopic pregnancy, women reported poor knowledge of EP (12). In India, although 25% of graduate-aged women and 43% of postgraduate-aged women reported being aware of EP, they demonstrated poor knowledge. Up to 98% had poor knowledge related to risk factors for EP (13). On the other hand, a study in southeastern Nigeria reported that up to 60% of participants were aware of EP, and the majority had a moderate to good level of knowledge about EP (14, 15).

Awareness of ectopic pregnancy is important, as unfavorable clinical outcomes are more likely to occur with poorer levels of EP knowledge (16). Furthermore, inadequate knowledge is associated with older age groups, lower-income groups, non-professional occupations, and lower educational attainment (16). Nurses, midwives, and other healthcare providers play a crucial role in educating women and their partners about the warning signs and symptoms of ectopic pregnancy, as well as providing preconception care. When an ectopic pregnancy is suspected, both the woman and her partner must be informed about the potential implications for her health and future fertility (17). Currently, there is limited research on women's knowledge of ectopic pregnancy in Saudi Arabia, especially in the central regions of Saudi Arabia. Also, there is limited evidence from Saudi Arabia assessing women's knowledge of ectopic pregnancy. This study was designed to help bridge the gap between theory and clinical practice. Enhancing awareness of potential risk factors for ectopic pregnancy can facilitate early detection, reduce morbidity, and prevent complications. Therefore, prioritizing early recognition and timely treatment is critical to minimizing the adverse outcomes and mortality associated with ectopic pregnancy.

## 2 Materials and methods

### 2.1 Study design

A descriptive cross-sectional study design was utilized to assess the level of knowledge about ectopic pregnancy among married Saudi women.

### 2.2 Aim of the study

This study aimed to assess the level of knowledge related to ectopic pregnancy among married Saudi women.

### 2.3 Research questions

1. What is the level of knowledge about ectopic pregnancy among married Saudi women?
2. How does the level of knowledge about ectopic pregnancy among married Saudi women vary based on demographic status and obstetrical variables?

### 2.4 Sample

A convenience sample of approximately 255 women visited the outpatient gynecologic and obstetric clinic at the Women's Health Specialist Hospital at King Abdulaziz Medical City, which was selected using a convenience sampling approach due to its accessibility and high patient volume. Data Collection was conducted over a 3-month period from early January 2024 to the end of March 2024. The study sample was recruited according to the following inclusion criteria: Married Saudi women aged 18–45 years. Women who attended any educational sessions regarding ectopic pregnancy in the last 12 months or had experienced an ectopic pregnancy were excluded from the current study.

### 2.5 Sample size

The sample size was calculated using *Raosoft*, a software tool that determines the necessary sample size for the current study based on the average number of patients (approximately 15,000 patients per year), with a 95% confidence level and a 0.5% confidence interval. The calculated sample size was 248, and a sample of 255 was included in the study to ensure the representativeness of the total population.

### 2.6 Setting

The study was conducted at the outpatient clinic for prenatal care at the National Guard Women's Health Hospital, Riyadh, Saudi Arabia. It is an affiliated hospital that offers a comprehensive range of services, including labor and delivery, C-section surgery, and gynecological procedures, as well as intensive and intermediate care for neonates. It includes an *in vitro* fertilization clinic, a maternal-fetal medicine clinic, a breast health clinic, a general obstetrics and gynecology clinic, and a comprehensive imaging department with a capacity of 300 beds.

### 2.7 Tools of data collection

The questionnaire was constructed by the researchers after an extensive review of the literature (18), and it was reviewed by a panel of maternity nursing professors at the King Saud bin Abdulaziz University for Health Science. This tool was written in simple and straightforward Arabic. This study aimed to assess maternity women's knowledge regarding ectopic pregnancy and included three parts. The first part included sociodemographic data, such as women's age, educational level, and marital status. The second part included gynecological and obstetrical data, such as menstrual regularity, number of pregnancies, number of deliveries, number of abortions, date of the last labor, complications in previous pregnancies, and current pregnancy history. The third part was divided into five sections, including a knowledge assessment sheet concerning women's knowledge and attitudes regarding ectopic pregnancy. It covered information about the definition, types, causes, and risk factors for ectopic pregnancy (from questions 1 to 7). Part two included knowledge of women regarding the signs and symptoms of ectopic pregnancy (from questions 8 to 9). Part three examined the knowledge of women regarding the diagnosis, management, and treatment of ectopic pregnancy (from questions 10 to 11). Part four included women's knowledge regarding the management of ectopic pregnancy (from questions 12 to 13). Part five assessed the knowledge of women regarding complications (from questions 14 to 15), with a total of 15 questions. In the scoring system, each question was assigned a score of 1 for the incorrect answer or “I don’t know,” a score of 2 for the correct but incomplete answer, and a score of 3 for the correct and complete answer. The total knowledge score was classified as follows: correct knowledge (good) ≥75%, incomplete knowledge (fair) from 74% to 50%, and incorrect (poor) knowledge less than 50%.

### 2.8 Tool validity and reliability

The face validity and content validity of the questionnaire were developed by the researcher, and five experts in the field of maternity nursing and obstetric medicine tested its content validity. Modifications were made according to the experts' judgment on the clarity of the sentences and the appropriateness of the content. After the questionnaire was collected, the researcher applied test reliability to assess the internal consistency of the tool. Cronbach's alpha = *α* in part I was 0.72, that in part II was 0.77, that in part III was 0.85, that in part IV was 0.88, that in part V was 0.82, and the total tool reliability was estimated at 0.95, indicating a strong positive correlation between the tools.

### 2.9 Procedures

Over three months, beginning in early January 2024 and ending of March 2024, data were collected. Three days a week (Monday, Wednesday, and Thursday) from 9 AM to 12 PM, the researcher attended an outpatient obstetrics and gynecology clinic. After receiving official permission from the Women's Health Specialist Hospital at King Abdulaziz Medical City, the researcher contacted the medical and nursing directors of the outpatient gynecologic and obstetrics clinics to explain the study's purpose and benefits to the participants, all participants received a written description of the study before enrolling and written formal consent was obtained from all participants. The researcher then recruited the women from the outpatient obstetrics and gynecology clinic, explained the nature and goal of the study, and obtained informed consent from those who met the criteria for inclusion and agreed to participate. Every woman included in the study sample was interviewed in the waiting area to obtain information on her demographics, her obstetrical and gynecological health, and her level of knowledge about ectopic pregnancy. To collect data, the researcher spoke with each woman one-on-one privately, asked the questions in simple Arabic, and recorded each woman's response in a questionnaire. Each woman's interview lasted between ten and fifteen minutes.

### 2.10 Statistical design

To prevent any inconsistencies, the researcher reviewed all the data. The data were checked for coding and entry errors, and then the data were tabulated and presented via both inferential and descriptive statistics. The level of significance was set at <0.05, and *p* > 0.05 denotes a significant result. The *p*-value represents the likelihood of error in the conclusion.

## 3 Results

The study utilized descriptive methods to characterize the study sample. Chi-square analysis was employed to assess relationships within the data, with significance determined by a threshold of *p* < 0.05. The findings revealed no statistical relationships between demographics, obstetrical factors, or the level of knowledge regarding ectopic pregnancy.

The results of the current study are presented below.

### 3.1 Part (I): demographic characteristics of the study sample

Table 1 shows the frequency distributions of the demographic characteristics. Nearly half of the study sample (48%) were aged between 36 and 45 years. A total of 10.2% of them were aged between 18 and 25 years, with a mean age of 35.50 ± 6.45 years. Regarding the level of education, more than half of the study sample (66.7%) had a university education. However, 0.8% were unable to read and write. Most of the participants (92.9%) were married.

**Table 1:** Distribution of the study sample by demographic characteristics (*N* = 255).

Items	*N*	%
Age		
<18	0	0.0
18–25 years	26	10.2
26–35 years	81	31.8
36–45 years	148	48
Mean ± SD yrs	35.50 ± 6.45	
Educational level		
Can't read or write	2	0.8
Primary Education	10	3.9
Preparatory Education	20	7.8
Secondary School	53	20.8
University Education	170	66.7
Marital status		
Married	237	92.9
Divorced	18	7.1

Table 2 shows the frequency distribution of the obstetric history of the study sample. Two-thirds of the study participants (79.6%) had regular menstrual cycles. More than half of them (56.9% and 52.2%, respectively) had experienced more than three pregnancies and deliveries. Approximately 36.1% of the samples had undergone 1–2 abortions. Among the study participants, 63.9% delivered via normal vaginal delivery, whereas 27.8% delivered via cesarean section. Additionally, 48.6% of the sample reported using family planning methods, with 32.5% using oral contraceptives.

**Table 2:** Distribution of the study sample according to the obstetrical history (*N* = 255).

Items	Freq.	%
Menstrual cycle regularity		
Regular	203	79.6
Irregular	52	20.4
Number of pregnancies		
Nulliparous	12	4.7
1–2	71	27.8
3	27	10.6
More than 3	145	56.9
Number of deliveries		
Nulliparous	21	8.2
1–2	71	27.8
3	30	11.8
More than 3	133	52.2
Number of abortions		
Yes	113	44.31
No	142	55.7
1–2	92	36.1
3	12	4.7
More than 3	9	3.5
Mode of previous delivery		
Yes	234	91.76
No	21	8.2
Normal Vaginal delivery	163	63.9
Cesarean Section	71`	27.8
Family Planning Methods		
Yes	124	48.6
No	131	51.4
If yes		
Types of family planning methods: Oral contraceptives	83	32.5
IUD	29	11.4
Condom	12	4.7

### 3.2 Part (II): description of the study sample toward the level of knowledge related to ectopic pregnancy among married Saudi women

**Figure 1:**
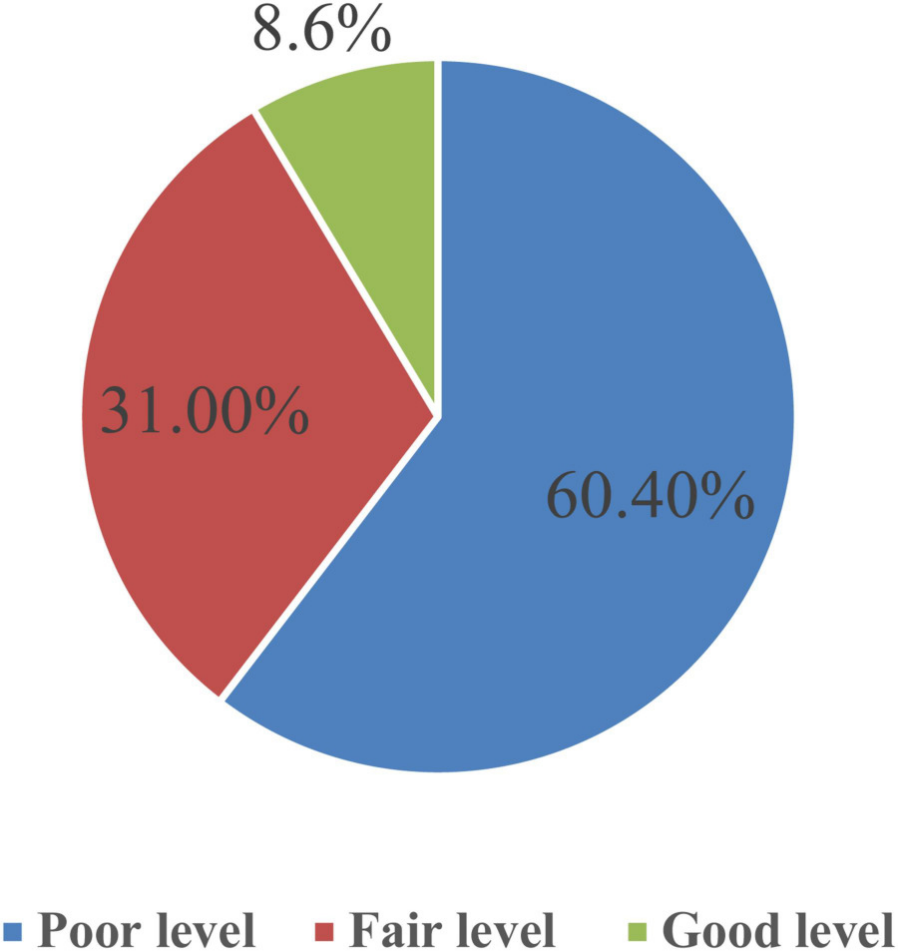
Distribution of the study sample according to the level of knowledge of ectopic pregnancy.

Table 3 presents the study sample's understanding of ectopic pregnancy, including its definition, causes, risk factors, signs and symptoms, complications, and management, with a mean knowledge score of 56.96 ± 17.09. Among the total knowledge categories, more than half of them had a poor level of knowledge, 31% had a fair level, and the remaining score of 8.6% had a good level of knowledge regarding ectopic pregnancy (Figure 1).

**Table 3:** Distribution of the study sample toward the level of knowledge (*N* = 255).

Variables	I don't know (1)		Incomplete Answer (2)		Complete Answer (3)	
	Freq.	%	Freq.	%	Freq.	%
Part I						
Definition of ectopic pregnancy	70	27.5	68	26.7	117	45.9
Types of ectopic pregnancy	171	67.1	50	19.6	34	13.3
Fallopian tube	150	58.8	44	17.3	61	23.9
ovaries	157	61.6	57	22.4	41	16.1
On isthmus	166	65.1	53	20.8	36	14.1
Others	203	79.6	32	12.5	20	7.8
Causes of ectopic pregnancy	190	74.5	46	18	19	7.5
Inflammation	197	77.3	43	16.9	15	5.9
Hormonal disturbance	161	63.1	72	28.2	22	8.6
Pelvic operation	190	74.5	44	17.3	21	8.2
Uterus anomalies	160	62.7	65	25.5	30	11.8
Risk factors for ectopic pregnancy	140	54.9	55	21.6	60	23.5
Previous ectopic	177	69.4	43	16.9	35	13.7
Infertility	188	73.7	44	17.3	23	9
Family history	184	72.2	43	16.9	28	11
Age less than 18 years	198	77.6	33	12.9	24	9.4
Exhaustion	187	73.3	47	18.4	21	8.2
Tubal ligation	162	63.5	58	22.7	35	13.7
Mean total score part I	26.55 ± 7.64					
Categorize						
Poor	153 (60%)					
Fair	77 (30.2%)					
Good	25 (9.8%)					
Part II						
Signs and symptoms of ectopic pregnancy	165	64.7	58	22.7	32	12.5
vaginal bleeding	143	56.1	58	22.7	54	21.2
Abdominal pain	132	51.8	66	25.9	57	22.4
Lower abdominal &back pain	131	51.4	61	23.9	63	24.7
Pain during urination	155	60.8	61	23.9	39	15.3
Upper shoulder pain	201	78.8	36	14.1	18	7.1
Mean Total Score Part II	9.39 ± 3.62					
Categorize						
Poor	141 (55.3%)					
Fair	55 (21.6%)					
Good	59 (23.1%)					
Part III						
Diagnosis of ectopic pregnancy	141	55.3	47	18.4	67	26.3
Ultrasound	117	45.9	49	19.2	89	34.9
Blood test to measure B-HCG level	150	58.8	62	24.3	43	16.9
Laparoscopy	145	56.9	57	22.4	53	20.8
Mean total score part III	6.81 ± 2.68					
Categorize						
Poor	128 (50.2%)					
Fair	48 (18.8%)					
Good	79 (31%)					
Part IV						
Management of ectopic pregnancy	172	67.5	41	16.1	42	16.5
Regular follow-up	143	56.1	55	21.6	57	22.5
Laparoscopy surgery	144	56.5	62	24.3	49	19.2
Treating according to the cases	140	54.9	56	22	59	23.1
Mean Total Score Part IV	6.46 ± 2.56					
Categorize						
Poor	137 (53.7%)					
Fair	64 (25.1%)					
Good	54 (21.2%)					
Part V						
Complications of ectopic pregnancy	151	59.2	62	24.3	42	16.5
severe bleeding	134	52.5	62	24.3	59	23.1
Vaginal infection	168	65.9	70	27.5	17	6.7
Abdominal pain& distention	142	55.7	72	28.2	41	16.1
Nausea and vomiting	148	58	72	28.2	35	13.7
Problems with the bladder & intestine	170	66.7	61	23.9	24	9.4
Mean total score part V	9.27 ± 3.46					
Categorize						
Poor	143 (56.1%)					
Fair	59 (23.1%)					
Good	53 (20.8%)					
Mean total knowledge score	**56.96** **±** **17.09**					
Total knowledge categories						
Poor level	154 (60.4%)					
Fair level	79 (31%)					
Good level	22 (8.6%)					

**Figure 2:**
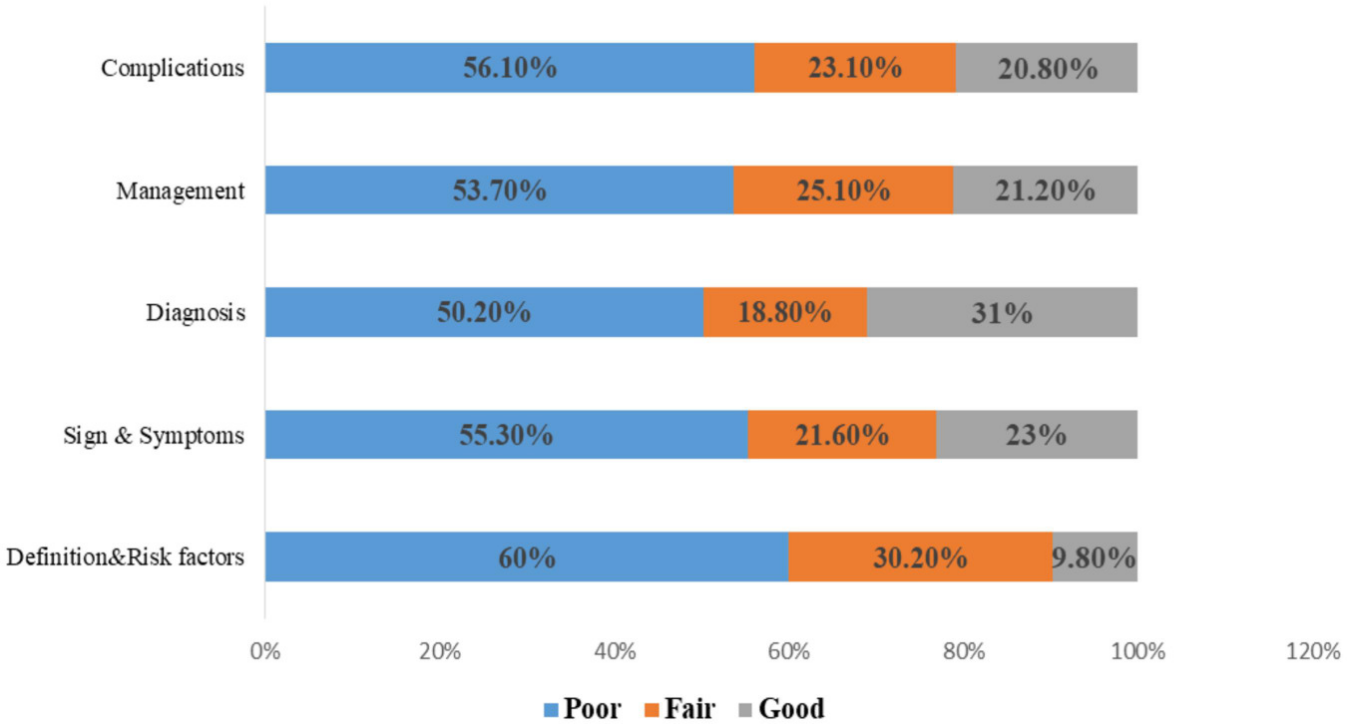
Distribution of the study sample regarding the categories of knowledge of ectopic pregnancy.

Specifically, among the participants in **part I**, 60% exhibited a poor level of knowledge concerning the definitions, types, causes, and risk factors associated with ectopic pregnancy. Conversely, 9.8% demonstrated a good level of understanding, whereas the remaining 30.2% possessed fair knowledge (Figure 2). The mean score obtained was 26.55, with a standard deviation of ±7.64. According to **Part II** of the study, the mean score recorded was 9.39, with a standard deviation of ±3.62. Concerning the understanding of signs and symptoms associated with ectopic pregnancy, 23.1% of the participants demonstrated a good level of knowledge, 21.6% exhibited fair understanding, and the majority, accounting for 55.3%, presented a poor level of knowledge. **Part III** revealed that the mean score for knowledge of diagnostic tests for ectopic pregnancy was 6.81, with a standard deviation of ±2.68. Among the participants, 50.2% exhibited poor knowledge, 18.8% demonstrated fair understanding, and 31% showed good comprehension of the diagnostic criteria of ectopic. Among the participants in **part IV**, 53.7% lacked knowledge regarding the management of ectopic pregnancy, 21.2% demonstrated a good understanding, and 25.1% exhibited fair knowledge. The mean score for this section was recorded as 6.46, with a standard deviation of ±2.56. Finally, in **part V** of the study, 56.1% of the participants had a poor understanding of the complications associated with ectopic pregnancy, 23.1% had fair knowledge, and 20.8% had a good level of understanding. The mean score for this section was calculated as 9.27, with a standard deviation of ±3.46.

As shown in the figure, in terms of overall knowledge categories, over half (60.40%) reported a low level of understanding, 31% reported a moderate level, and the remaining 8.6% reported a high level of knowledge concerning ectopic pregnancy.

Table 4 shows a summary of the minimum, maximum, mean, and standard deviation of all the knowledge regarding ectopic pregnancy among the study sample.

**Table 4:** Distribution of the study sample toward a summary of scores regarding knowledge of ectopic pregnancy (*N* = 255).

Variables	Minimum	Maximum	M	±SD
Part I	18	54	26.55	7.64
Part II	6	18	9.39	3.62
Part III	4	12	6.81	2.68
Part IV	4	12	6.46	2.56
Part V	6	18	9.27	3.46
Total knowledge	38	114	56.96	17.09

### 3.3 Part (III): the relationship between selected demographic & obstetrical variables and level of knowledge toward ectopic pregnancy

Table 5 shows that there was no relationship between selected demographic variables, such as maternal age, educational level, marital status, and level of knowledge of ectopic pregnancy, with *p* values >0.05.

**Table 5:** Relationships between selected demographics and level of knowledge of ectopic pregnancy (*N* = 255).

Items	Poor (*n* = 154)	Fair (*n* = 79)	Good (*n* = 22)	*t* test
Age				
18–25 years	14 (9.1)	7 (8.9)	5 (22.7)	X = 6.49,*p* = 0.16
26–35 years	47 (30.5)	30 (38)	4 (18.2)	
36–45 years	93 (60.4)	42 (53.2)	13 (59.1)	
Educational level				
Can't read or write	2 (1.3)	0 (0.0)	0 (0.0)	X = 8.05,*p* = 0.42
Primary Education	7 (4.5)	1 (1.3)	2 (9.1)	
Preparatory Education	11 (7.1)	8 (10.1)	1 (4.5)	
Secondary School	32 (20.8)	16 (20.3)	5 (22.7)	
University Education	102 (66.2)	54 (68.	14 (63.6)	
Marital status				
Married	147 (95.5)	70 (88.6)	20 (90.9)	X = 3.88,*p* = 0.14
Divorced	7(4.5)	9(11.4)	2(9.1)	

Table 6 shows that there was no relationship between selected obstetrical variables, such as maternal menstrual cycle regularity, number of pregnancies, number of deliveries, number of abortions, mode of previous delivery, and used family planning methods, and level of knowledge toward ectopic pregnancy, with a *p* value >0,05.

**Table 6:** Relationships between obstetrical variables and level of knowledge of ectopic pregnancy (*N* = 255).

Items	Poor (*n* = 154)	Fair (*n* = 79)	Good (*n* = 22)	*T* test
Menstrual cycle regularity				
Regular	123 (79.9)	63 (79.7)	17 (77.3)	X = 0.08,*p* = 0.96
Irregular	31 (20.1)	16 (20.3)	5 (22.7)	
Number of pregnancies				
Nulliparity	6 (3.9)	4 (5.1)	2 (9.1)	X = 6.42,*p* = 0.37
1–2	38 (24.7)	45 (57)	7 (31.8)	
3	20 (13)	4 (5.1)	3 (13.6)	
More than 3	90 (58.4)	26 (32.9)	10 (45.5)	
Number of deliveries				
Nulliparous	13 (8.4)	9 (9)	2 (8.7)	X = 2.58,*p* = 0.85
1–2	39 (25.3)	30 (30)	9 (39.1)	
3	20 (13)	10 (10)	3 (13)	
More than 3	82 (53.2)	51 (51)	9 (39.1)	
Number of abortions				
No	6 (3.9)	5 (6.3)	1 (4.5)	X = 2.71,*p* = 0.84
1–2	5 (3.2)	4 (5.1)	0 (0.0)	
3	85 (55.2)	45 (57)	12 (54.5)	
More than 3	58 (37.7)	25 (31.6)	9 (40.9)	
Mode of previous delivery				
No	14 (9.1)	4 (5.1)	3 (13.6)	X = 4.72,*p* = 0.31
Normal Vaginal delivery	93 (60.4)	54 (68.4)	16 (72.7)	
Cesarean Section	47 (30.5)	21 (26.6)	3 (13.6)	
Family Planning Methods				
Yes	72 (46.8)	39 (49.4)	13 (59.1)	X = 1.19,*p* = 0.54
No	82(53.2)	40(50.6)	9(40.9)	

Regarding the definition and risk factors, 60% of the participants exhibited a poor level of knowledge, 30.20% demonstrated fair knowledge, and 9.80% possessed good knowledge. Concerning the signs and symptoms of ectopic pregnancy, 55.30% had poor knowledge, 23% demonstrated good knowledge, and 21.60% had fair knowledge. In terms of diagnosis, 5.20% displayed a good understanding, 18.80% had fair knowledge, and 31% possessed good knowledge. Concerning management, 21.20% exhibited good knowledge, whereas the majority, comprising 53.70%, had poor knowledge, and 25.10% had fair knowledge. Finally, concerning the complications of ectopic pregnancy, the majority, 56.10% of the sample, demonstrated a poor level of knowledge, 23.10% had fair knowledge, and 20.80% exhibited good knowledge.

## 4 Discussion

This study sought to evaluate awareness levels regarding ectopic pregnancy (EP) among married Saudi women in Riyadh. Our demographic analysis revealed a significant concentration of participants within the 36–45 years age bracket, with a notable representation from the 26–35 years age group. The majority of participants had attained a university education, indicating a relatively educated cohort. Notably, the variation in the age and education level of the participants did not impact their level of awareness of EP. Furthermore, the study sample predominantly comprised married women, thus providing focused insight into this demographic segment's awareness levels.

Regarding knowledge levels, our findings revealed that more than half of the participants exhibited a limited understanding of EP, whereas approximately one-third demonstrated a fair understanding. These results were compared with those of a similar study focusing on married women from Chi Linh district in Vietnam (19), which reported comparable levels of inadequate knowledge. Notably, a study focused on younger participants from Southeastern Nigeria aged 20–24 years has reported a higher prevalence of good knowledge, suggesting potential age-related disparities in awareness levels (15). Also, up to 98% reported poor knowledge related to the signs, symptoms, and risk factors of ectopic pregnancy (13). Based on our analysis, the difference between our study and a similar study can be attributed to the targeted age groups (15). We focused on older women, whereas the other study in Southeastern Nigeria targeted younger women (15). This age difference likely contributed to the positive results observed in the study, as younger participants may have better access to information and educational resources regarding EP. However, a hospital-based study in Pakistan documented how often specific signs/symptoms occur among patients with ectopic pregnancy: amenorrhea (87.5%), abdominal pain (81.3%), vaginal bleeding (68.8%) were among the most frequent. Yet, such evidence underscores how common these symptoms are in EP, highlighting the gap between public awareness and what is clinically typical (20).

Exploring EP risk factors, our study underscored a prevalent lack of knowledge among more than half of the participants. This aligns with findings from the study in India, which emphasized a widespread deficit in understanding EP risk factors among married women (13). Contrary outcomes were reported in a study that was conducted among female undergraduates in southeastern Nigeria (19). The study observed a higher level of knowledge, highlighting the influence of educational background and age on variations in knowledge (19).

Concerning signs and symptom comprehension, our study revealed a substantial knowledge gap, with more than half of the participants exhibiting poor awareness; they did not know that bleeding, abdominal pain, lower back pain, and shoulder pain were signs of EP. A similar trend was reported, indicating a significant lack of understanding of signs and symptoms among participants, where most participants knew only one sign of abnormal pregnancy (13). Nearly half of the participants demonstrated good knowledge of signs and symptoms, suggesting that potential variation in the targeted sample can contribute to the variation in the level of awareness (19). Thus, a lack of awareness of ectopic pregnancy symptoms can lead to delayed diagnosis and mismanagement, which increases the risk of life-threatening complications. Improved education and awareness campaigns are crucial for promoting early detection, effective management, and better outcomes for individuals affected by EP.

The observed variability in knowledge levels across participants, despite similarities in marital status and relatively high educational attainment, suggests substantial heterogeneity within the study population. This heterogeneity may reflect unmeasured factors such as prior healthcare experiences, *in vitro* fertilization treatment, or interaction with healthcare services. High heterogeneity limits the generalizability of aggregate knowledge scores, indicating that awareness gaps are not confined to specific demographic subgroups. Consequently, uniform educational strategies may be insufficient, and tailored awareness programs addressing diverse informational needs are warranted.

Despite thorough consideration of demographic variables such as maternal age, educational attainment, and marital status, our study did not establish a significant correlation between the level of knowledge about EP and these variables. This finding, which aligns with previous studies (15), further emphasizes that heterogeneity in awareness exists beyond conventional demographic predictors. Therefore, comprehensive public health strategies that deliver consistent, accessible, and culturally appropriate education on ectopic pregnancy are essential to improve early recognition, prompt care-seeking behavior, and maternal health outcomes.

## 5 Conclusion

In summary, the findings of this study highlight a concerning trend regarding knowledge levels surrounding ectopic pregnancy, with a significant majority of the sample demonstrating a poor understanding. With only a minority possessing a fair or good level of knowledge, there is a pressing need for targeted education and awareness campaigns to improve understanding and ultimately reduce the risks associated with this potentially life-threatening condition. Clinically, inadequate awareness of ectopic pregnancy may contribute to delayed presentation, misinterpretation of symptoms, and increased risk of complications, including tubal rupture and maternal morbidity. Enhancing public knowledge can facilitate earlier recognition of warning signs, prompt medical evaluation, and timely intervention, ultimately improving maternal health outcomes.

## Data Availability

The raw data supporting the conclusions of this article will be made available by the authors, without undue reservation.

## References

[1] Mullany K Minneci M Monjazeb R C. Coiado O. Overview of ectopic pregnancy diagnosis, management, and innovation. Womens Health. (2023) 19:17455057231160349. 10.1177/17455057231160349 PMC10071153 36999281

[2] Vadakekut ES Gnugnoli DM. Ectopic Pregnancy. in: StatPearls. Treasure Island (FL): StatPearls Publishing (2025). Available online at: http://www.ncbi.nlm.nih.gov/books/NBK539860/ (Accessed September 21, 2025). 30969682

[3] Gerema U Alemayehu T Chane G Desta D Diriba A. Determinants of ectopic pregnancy among pregnant women attending referral hospitals in southwestern part of Oromia regional state, Southwest Ethiopia: a multi-center case control study. BMC Pregnancy Childbirth. (2021) 21(1):130. 10.1186/s12884-021-03618-7 33579224 PMC7881641

[4] Sivalingam VN Duncan WC Kirk E Shephard LA Horne AW. Diagnosis and management of ectopic pregnancy. J Fam Plan Reprod Health Care Fac Fam Plan Reprod Health Care R Coll Obstet Gynaecol. (2011) 37(4):231–40. 10.1136/jfprhc-2011-0073 PMC3213855 21727242

[5] Stabile G Vona L Carlucci S Pitsillidi A Restaino S Vizzielli G Uterine ectopic pregnancies and live births: systematic review of the literature and concepts underlying favorable outcomes. Medicina (Mex). (2025) 61(11):1915. 10.3390/medicina61111915 PMC12653997 41303752

[6] Papakonstantinou A Moustakli E Potiris A Zikopoulos A Tsarna E Christodoulaki C Behind-the-Scenes actors in fertility: a comprehensive review of the female reproductive tract microbiome and its clinical relevance. Life. (2025) 15(6):916. 10.3390/life15060916 40566568 PMC12193755

[7] McQueen BE Kiatthanapaiboon A Fulcher ML Lam M Patton K Powell E Human fallopian tube epithelial cell culture model to study host responses to Chlamydia trachomatis infection. Infect Immun. (2020) 88(9):10.1128/iai.00105-20. 10.1128/IAI.00105-20 PMC7440757 32601108

[8] American College of Obstetricians and Gynecologists. ACOG Practice bulletin No. 193: tubal ectopic pregnancy. Obstet Gynecol. (2018) 131(3):e91. 10.1097/AOG.0000000000002560 29470343

[9] Richardson A Gallos I Dobson S Campbell BK Coomarasamy A Raine-Fenning N. Accuracy of first-trimester ultrasound in diagnosis of tubal ectopic pregnancy in the absence of an obvious extrauterine embryo: systematic review and meta-analysis. Ultrasound Obstet Gynecol. (2016) 47(1):28–37. 10.1002/uog.14844 25766776

[10] Hendriks E Rosenberg R Prine L. Ectopic pregnancy: diagnosis and management. Am Fam Physician. (2020) 101(10):599–606. PMID: 32412215. 32412215

[11] Raine-Bennett T Fassett MJ Chandra M Armstrong MA Xie F Shi JM Disparities in the incidence of ectopic pregnancy in a large health care system in California, 2010–2019. Perm J. (2022) 26(3):61–8. 10.7812/TPP/21.099 35939627 PMC9683753

[12] Zain H Albarakaty R Mohamed Y Abdallah Y. Clinical analysis of ectopic pregnancies in Majmaah, Saudi Arabia. Biomed Res. (2019) 30(5):800–4. 10.35841/biomedicalresearch.30-19-200 Available online at: https://www.alliedacademies.org/abstract/clinical-analysis-of-ectopic-pregnancies-in-majmaah-saudi-arabia-11422.html (Accessed September 24, 2025).

[13] Mehta (Pt) DrVA, Contractor (Pt) DrG. Knowledge of ectopic pregnancy in young adult females. Int J Health Sci Res. (2022) 12(11):282–6. 10.52403/ijhsr.20221136

[14] Chong KY de Waard L Oza M van Wely M Jurkovic D Memtsa M Ectopic pregnancy. Nat Rev Dis Primer. (2024) 10(1):94. 10.1038/s41572-024-00579-x 39668167

[15] Esu E Okpon EI. Knowledge of risk factors for ectopic pregnancy among female undergraduates in Southeastern Nigeria. Calabar J Health Sci. (2020) 4(1):8–12. 10.25259/CJHS_15_2020

[16] Smart G Tai A Wong JC Oliver R Odejinmi F. Social prevalence of knowledge about ectopic pregnancy—tip of the “health inequalities” iceberg? J Obstet Gynaecol. (2021) 41(3):428–33. 10.1080/01443615.2020.1741521 32515631

[17] Gyamtsho S Tenzin K Bhutia PC Tshomo T Choeda T. Assessment of knowledge, attitude, and practice on preconception care among pregnant women at national hospital, Thimphu, Bhutan. Bhutan Health J. (2022) 8(1):1–7. 10.47811/bhj.130

[18] Al-Musa HM Alsaleem MA Alfaifi WH Alshumrani Z Alzuheri NS Aslouf AS Knowledge, attitude, and practice among Saudi primary health care attendees about family planning in Abha, Kingdom of Saudi Arabia. J Fam Med Prim Care. (2019) 8(2):576–82. 10.4103/jfmpc.jfmpc_363_18 PMC6436269 30984676

[19] Bui HTT. Knowledge, attitude and behavior on ectopic pregnancy of married women in Chi Linh, Hai Duong. J Prev Med. (2008) 44–9.

[20] Qayyum H Wahab S. Frequency of common signs and symptoms of ectopic pregnancy. Insights-J Health Rehabil. (2025) 3(3):9–15. 10.71000/3vp56j57

